# Case report: Pemigatinib-induced retinopathy: a serial examination of subretinal fluid secondary to an FGFR inhibitor

**DOI:** 10.3389/fopht.2023.1247296

**Published:** 2024-01-22

**Authors:** Daniel Barmas-Alamdari, George Jiao, Ronni Lieberman

**Affiliations:** ^1^ Department of Ophthalmology, Northwell Health, New York, NY, United States; ^2^ Department of Ophthalmology, Mount Sinai, New York, NY, United States

**Keywords:** pemigatinib, subretinal fluid, FGFR (fibroblast growth factor receptor), FGFR inhibitor, pigment epithelial detachment, Pemazyre, OCT

## Abstract

**Background:**

Modern chemotherapeutic agents continue to evolve as modern monoclonal antibody treatments are designed to directly target proteins, enzymes, and focal loci. A particular class of these medications, fibroblast growth factor (FGFR) inhibitors, specifically pemigatinib (Pemazyre^®^; Incyte), has been approved by the US Food and Drug Administration since April 2020 for the treatment of advanced or metastatic cholangiocarcinoma. As it is a relatively new medication, its side-effect profile is manifesting in active clinical practice. The presence of FGFR receptors in the retinal pigment epithelium makes the retina susceptible to potential adverse effects secondary to pemigatinib use.

**Case presentation:**

A 69-year-old African-American male with a tumor mutation burden 3 (TMB-3) metastatic adenocarcinoma of the liver from primary cholangiocarcinoma, who was undergoing chemotherapy with pemigatinib, was found to have asymptomatic bilateral subretinal fluid accumulation. Serial monitoring with optical coherence tomography (OCT) demonstrated complete resolution of the subretinal fluid while off-cycle and asymptomatic re-accumulation of subretinal fluid while on-cycle, with no significant changes in visual acuity.

**Conclusions:**

Subretinal fluid accumulation secondary to pemigatinib may develop during the active treatment cycles without causing any significant visual symptoms for the patient. Serial monitoring demonstrates fluctuations of subretinal fluid during the patient’s on- and off-cycles. This case strengthens the current guidelines for continuing pemigatinib in asymptomatic patients found to have subretinal fluid. Further studies are warranted to identify patients who may be at higher risk for developing subretinal fluid.

## Background

Modern chemotherapeutic agents continue to evolve as monoclonal antibody treatments directly targeting proteins, enzymes, and focal loci become standards of treatment. A particular class of these medications, fibroblast growth factor receptor (FGFR) inhibitors, specifically pemigatinib (Pemazyre^®^; Incyte), has been approved by the US Food and Drug Administration since April 2020 for the treatment of advanced or metastatic cholangiocarcinoma. As it is a relatively new medication, its side-effect profile is only now becoming more evident. Serous retinopathy is a known complication of FGFR inhibitors (1–4). In clinical trials, retinal pigment epithelial detachment (RPED), secondary to serous retinopathy, occurred in 11% of patients, with 1.3% having grade 3 or 4 RPED (5). The presence of FGFRs in the retinal pigment epithelium makes the retina susceptible to this potentially adverse effect of pemigatinib. The current guidelines, based on clinical trials, recommend optical coherence tomography (OCT) testing prior to initiating therapy and every 2 months for the first 6 months of the treatment, followed thereafter by every 3 months. If patients are asymptomatic, the recommendation is to continue the medication. If symptoms develop or worsen, it is suggested to withhold pemigatinib and have a repeat examination to determine whether the drug should be withheld permanently or whether it could be continued at a lower dose. In the trials, only 3.1% of patients required dose interruption; 1.3% required dose reduction, and 0.2% permanently discontinued the treatment. Importantly, however, routine monitoring with OCT was not conducted in the clinical trials to detect asymptomatic RPED or subretinal fluid (SRF) and, therefore, incidence of these are unknown. We sought to follow a patient on a stable regimen of pemigatinib, and to demonstrate that fluctuations in SRF accumulation may not correlate with visual symptoms. This evidence would aid clinicians in determining treatment dosage and frequency adjustments.

## Case presentation

**Figure 1:**
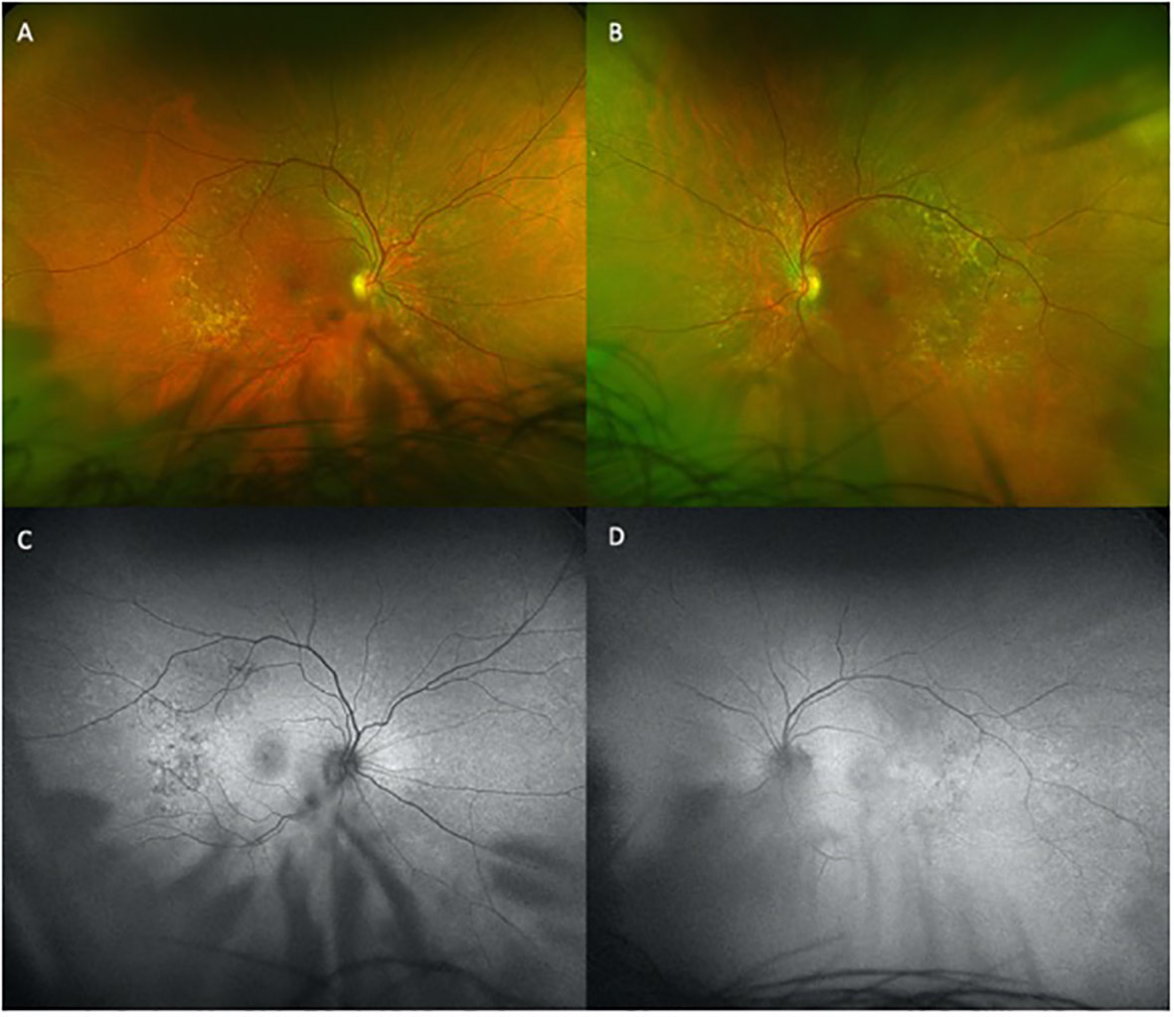
Temporal and nasal macular drusen demonstrated on fundus photography and autofluorescence, presenting on baseline examination. Hypopigmentation showing drusen present inferiorly. Limited by patient’s cataracts. **(A)** and **(B)** are the fundus photos for the right and left eye respectively. **(C)** and **(D)** are the autofluorescence images for the right and left eye respectively.

A 69-year-old African-American male presented for baseline ophthalmic examination prior to beginning pemigatinib treatment. His medical history was significant for a tumor mutation burden 3 (TMB-3) metastatic adenocarcinoma of the liver, which was associated with bilateral pulmonary metastases arising from primary cholangiocarcinoma. Other significant medical history characteristics included well-controlled type 2 diabetes mellitus and hypertension. The patient denied having any visual complaints at this stage. The ophthalmic history included stable peripheral drusen, orbital fat prolapse, and mild cataracts in both eyes. The initial ophthalmic examination revealed a non-corrected visual acuity (VA) of 20/40 in the right eye (OD; oculus dexter) and 20/20−2 in the left eye (OS; oculus sinister). The best corrected VA OD was 20/30 at this time. The anterior examination was significant for mild dry eye and mild cataract bilaterally. The complete dilated fundus examination was significant for peripheral drusen. No RPED or SRF was noted on OCT testing. Baseline fundus autofluorescence (FAF) showed multiple areas of hypopigmented lesions consistent with the patient’s known temporal macular drusen, as seen in the color fundus photography (**Figure 1**).

**Figure 2:**
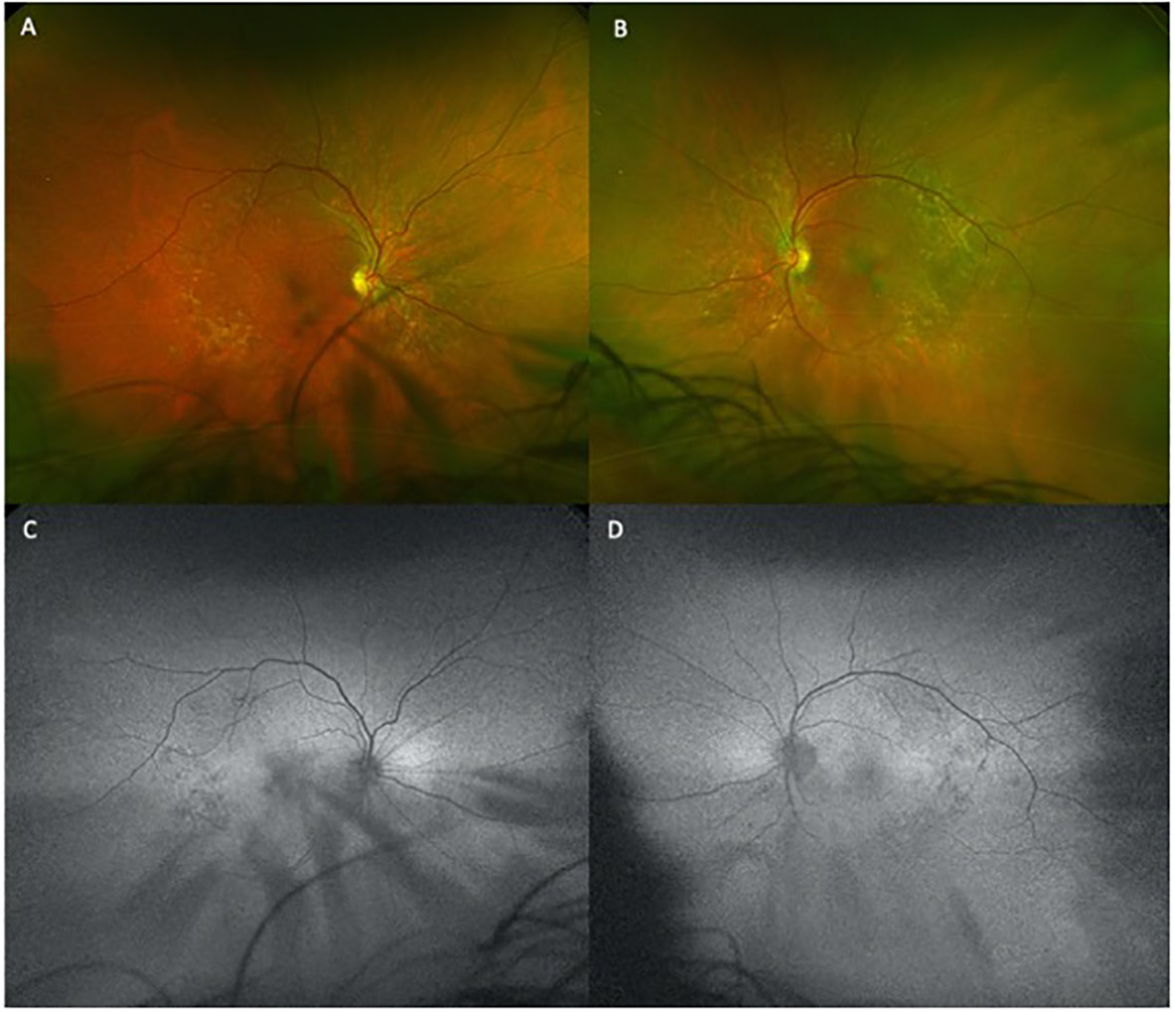
Temporal and macular drusen redemonstrated on fundus photography, with right eye exhibiting more hypopigmentation foveally on FAF and possible elevation bilaterally due to subretinal fluid. The changes are subtle and somewhat obscured. Questionable changes due to small amount of subretinal fluid accumulation prompted ceasing of autofluorescence imaging. **(A)** and **(B)** are the fundus photos for the right and left eye respectively. **(C)** and **(D)** are the autofluorescence images for the right and left eye respectively.

The patient began treatment with oral pemigatinib daily and was followed up for an ophthalmic examination during the second treatment cycle approximately 5 weeks into the treatment process. The patient had at this stage received 28 doses of 13.5 mg per day, which was administered in cycles of 14 on-days and 7 off-days, and had no visual or ophthalmic complaints. The non-corrected VA was OD 20/30 + 1 and OS 20/20. The OCT examinations revealed subfoveal SRF bilaterally. The fundus examination was otherwise unremarkable outside the patient’s prior documented peripheral drusen. Repeat autofluorescence was overall unchanged, though a slight hypofluorescent ring may be visualized in **Figure 2**. The current pemigatinib protocol recommendation for asymptomatic patients suggests no dose modification; however, with worsening presentation or positive symptoms, it is recommended to withhold pemigatinib. After discussion with the patient’s oncologist, it was decided to continue the medication at this time. Serial monitoring of the patient’s symptoms, vision, and SRF were conducted on specific days during active treatment cycles and days off-cycle to monitor the SRF. Subsequent evaluations demonstrated complete resolution of SRF while off-cycle, and asymptomatic re-accumulation of fluid while on-cycle with varying levels of VA. (**Table 1**) As we see demonstrated in the table below, in the later stages of the active cycles, such as day 13 of 14 of cycle 2, day 13 of 14 of cycle 3, day 13 of 14 of cycle 4, and day 14 of 14 on cycle 5, the patient’s VA showed no correlative changes based on the presence of SRF. The fluctuations in the patient’s VA certainly occurred, but were likely to be secondary to surface changes, as certain off-cycle days actually presented with lower acuities than days when the SRF was present on OCT examination.

**Table 1:** Highlights the visual acuity, central macular thickness, and the physical OCT of the macula for this patient in both the right and left eyes during various stages of the patient's chemotherapy treatment cycle.

Cycle, day, on/off	VA OD; OS	CMT OD; OS	OCT OD	OCT OS
Prior to initiation	20/40; 20/20−2	274; 275	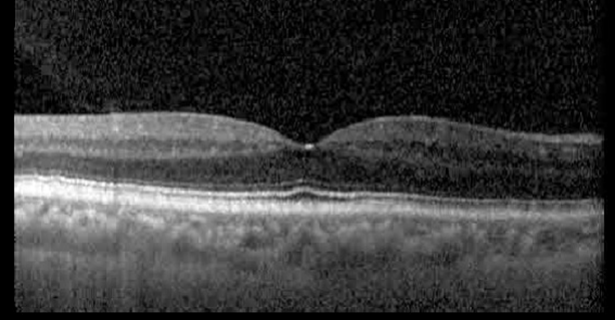	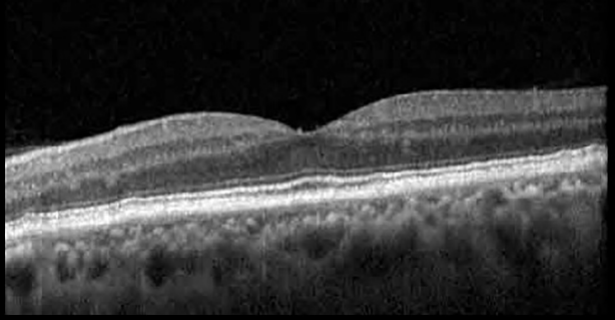
Cycle 2, day 13/14, on	20/30+1; 20/20	365; 355	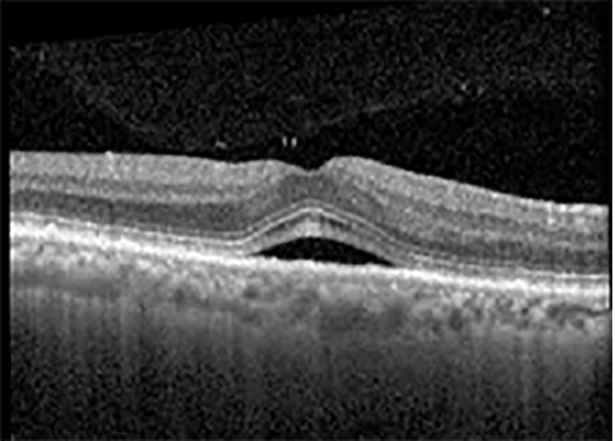	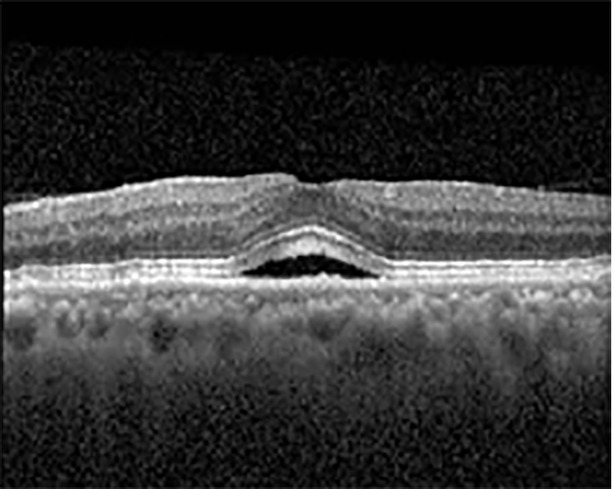
Cycle 2, day 4, off	20/30−2; 20/20−2	282; 280	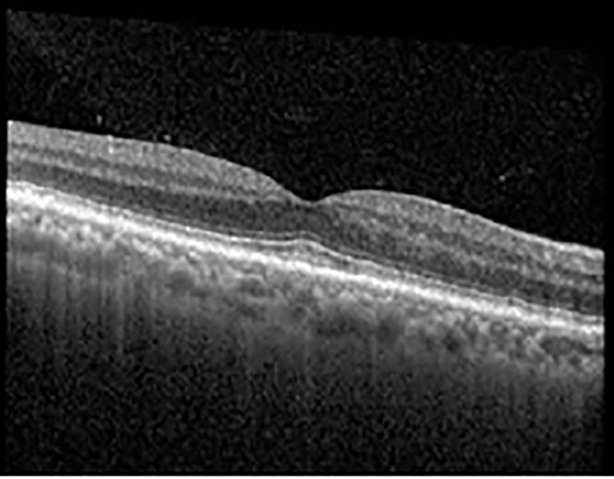	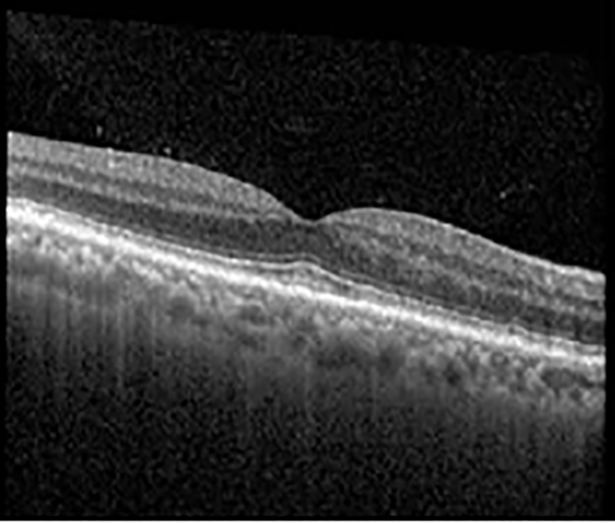
Cycle 3, day 6/14, on	20/40−1; 20/30−1	306; 301	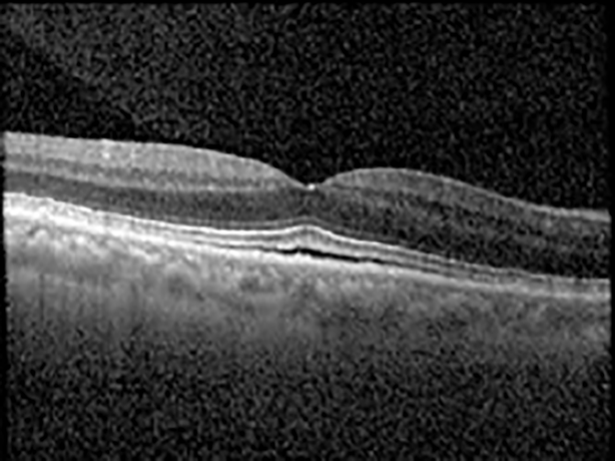	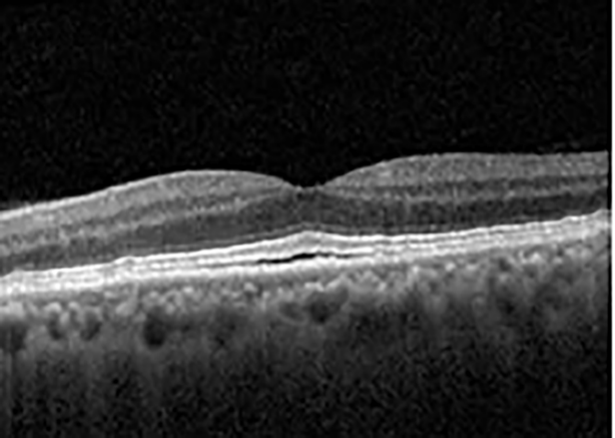
Cycle 3 day 13/14, Oon	20/50+1; 20/30−2	Poor tracing; 376	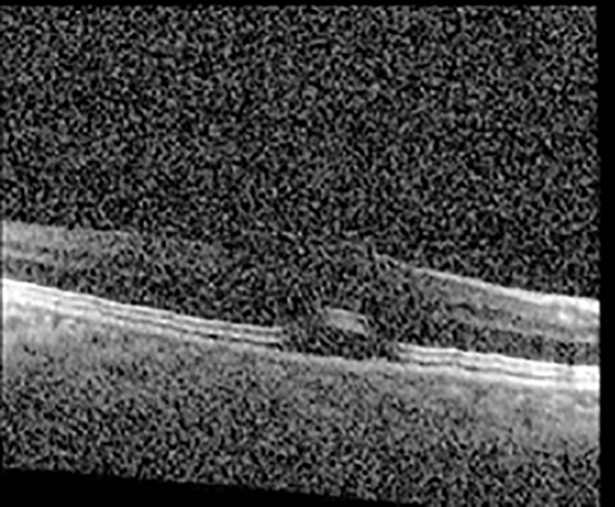	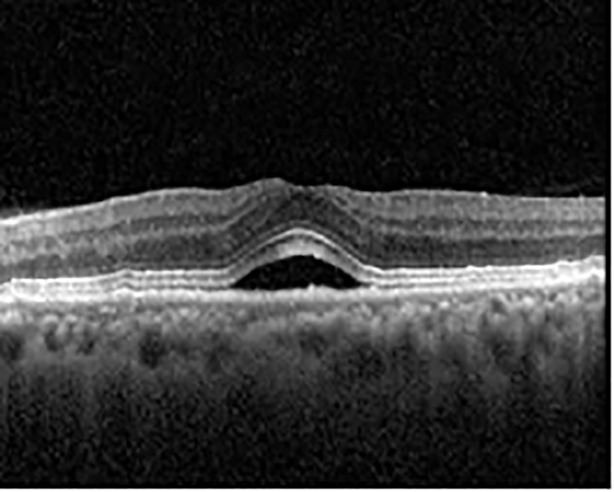
Cycle 3, day 3, off	20/30−1; 20/25	320; 329	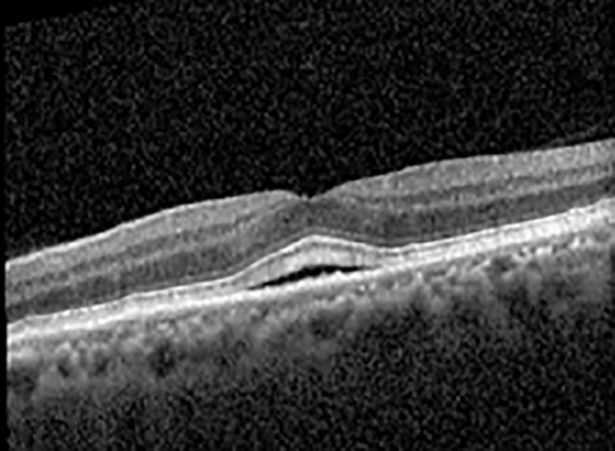	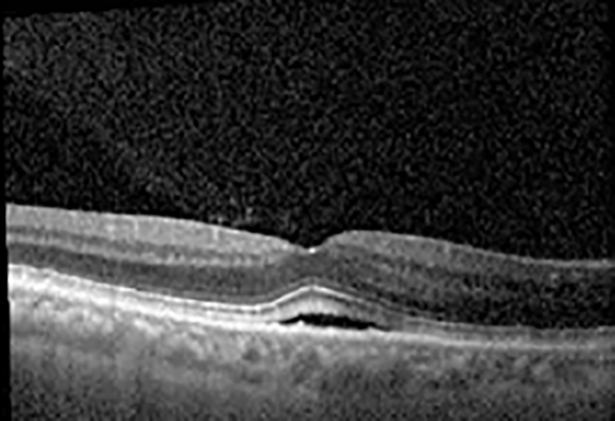
Cycle 3, day 10, off	20/50−2; 20/30−1	269; 276	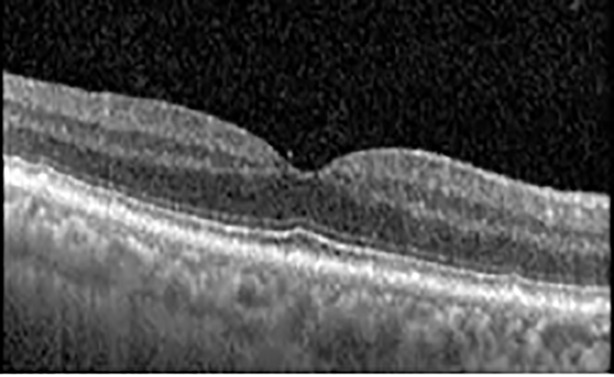	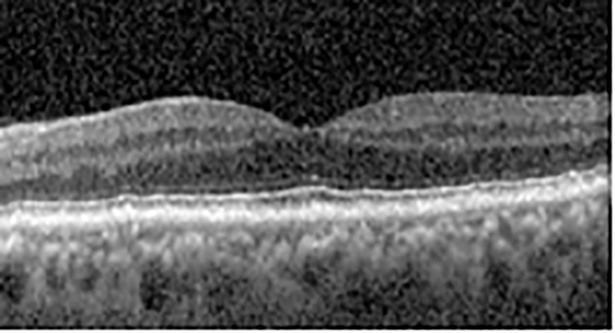
Cycle 4, day 8/14, on	20/50−2; 20/30	333; 317	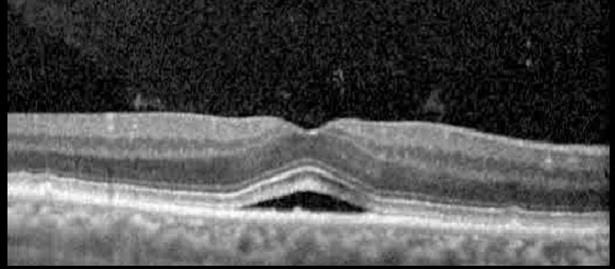	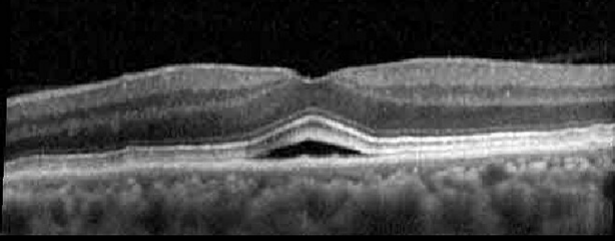
Cycle 4, day 13/14, on	20/40−2; 20/25	360; 348	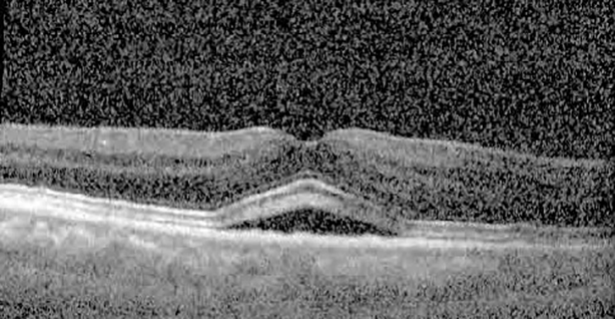	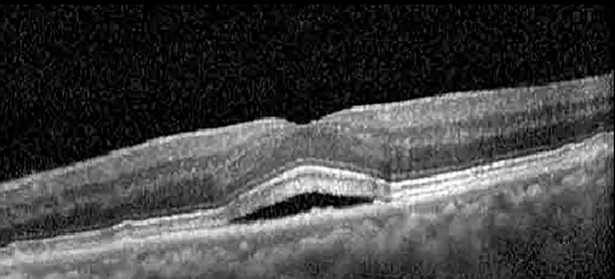
Cycle 4, day 1, off (9/6)	20/30; 20/25−1	273; 271	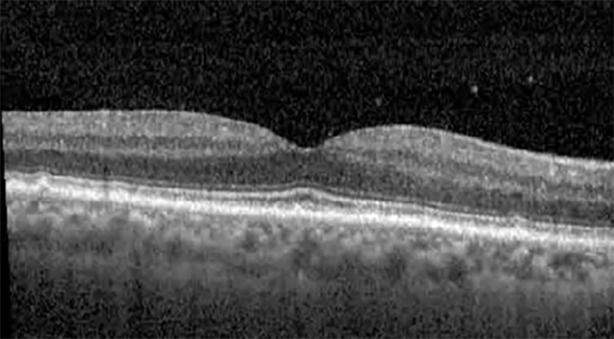	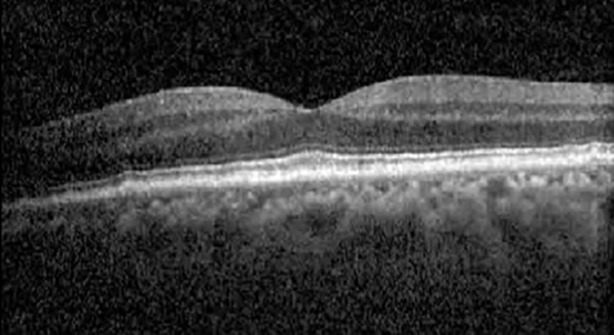
Cycle 5, day 14/14, on	20/30−1; 20/20	375; 364	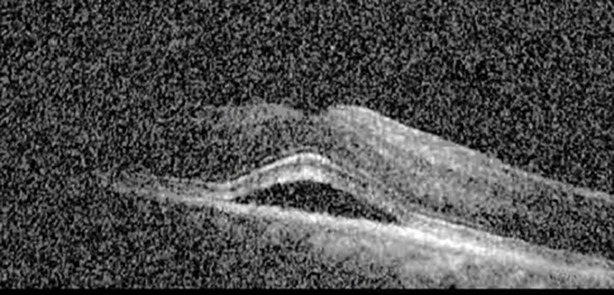	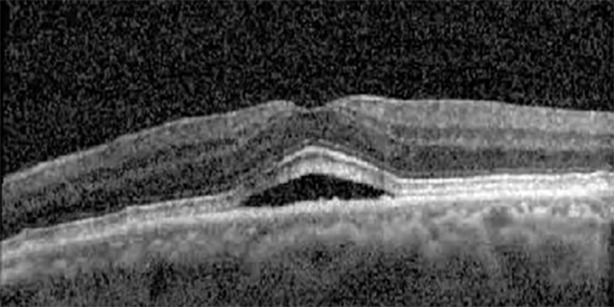
Cycle 5, day 6, off	20/40+2; 20/30−2	276; 278	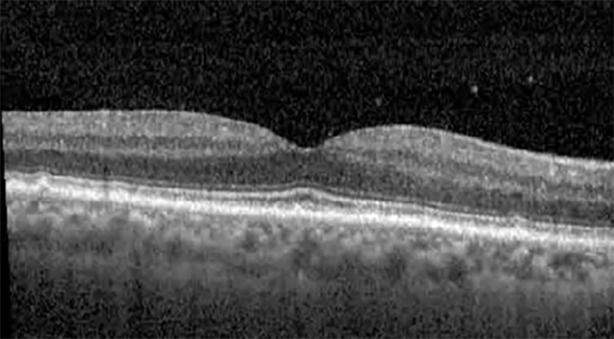	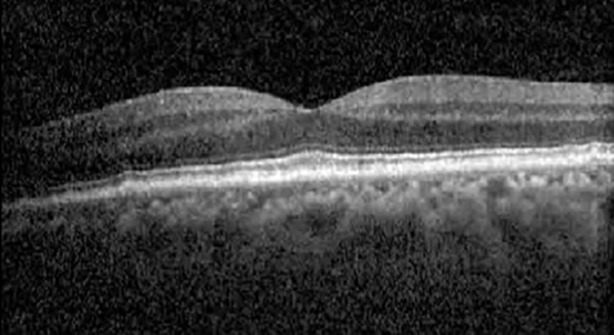

## Discussion

Pemigatinib is a chemotherapeutic medication in the class of FGFR inhibitors. The receptors targeted by the drug are tyrosine kinase receptors that serve to activate tumor signaling pathways (6). By inhibiting these downstream pathways, the medication restricts the activity of tumor cells that overproduce FGFRs. The medication is currently prescribed as an oral formulation in the treatment of cholangiocarcinoma: 13.5 mg daily for 14 consecutive days, followed by 7 days off-therapy (7).

FGF also serves an important role as a neurotrophic factor in the retinal pigment epithelium (8). It promotes growth in immature retinal pigment epithelium (RPE) cells and prevents the apoptosis of mature cells. Another factor in this signaling pathway that is activated downstream of FGF is mitogen-activated protein kinase (MAPK), which also serves to protect the RPE from injury (8). This provides a chemical link between FGFR inhibitors and MAPK inhibitors (MEK), which have previously been documented as causing SRF in the treatment of systemic cancers as a specific disease entity, i.e., MEK-induced retinopathy (8–10). Indeed, early cases show that other FGFR inhibitors, such as erdafitinib (Balversa^®^; Janssen Biotech) (1, 2), infigratinib (Truseltiq™; Helsinn Healthcare) (1) and AZD4547 (3) lead to retinopathy similar to that caused by MEK inhibitors. A case of pemigatinib-induced retinopathy was first described in 2021 by Aleekseev et al., with a transient multifocal SRF (11), and a subsequent case was described in 2022 by Bloom et al. (4).

During clinical trials for pemigatinib, routine ophthalmologic monitoring of patients’ retinal health was not conducted, thus the incidence of asymptomatic serous retinal detachments or development of SRF associated with pemigatinib is unknown. Currently, the guidelines for administering pemigatinib in patients found to have adverse effects are to continue the medication if the patient is asymptomatic and stable, withhold if symptomatic or worsening, resume at a lower dose if asymptomatic and improving, and discontinue if symptomatic and no improvement while being withheld (5). This case serves to demonstrate the fluctuation of SRF in an asymptomatic patient, which did not correlate with their VA, and strengthens the recommendation to continue chemotherapy in asymptomatic patients with SRF. It also serves to provide a framework for collaboration between oncologists and ophthalmologists to decide whether or not dosing changes are needed in asymptomatic patients, as our patient had no changes in treatment dosing yet maintained stable vision despite cyclical accumulations of SRF. Again, highlighting in the table, as above, the later days in each treatment cycle showed greater accumulations of SRF. However, importantly, the VA showed no demonstrable pattern of decline with the presence of SRF. We also see no significant changes on fundus photography or autofluorescence, suggesting that, at least in this case, OCT of the macula would be the only surefire way to detect these subtle changes. We see multiple times when fluid is present and VA is actually better than on the days when there was no presence of SRF. We believe this to be indicative of the fact that the SRF accumulation due to pemigatinib does not influence the patient’s acuity and thus, so long as the patient remains asymptomatic throughout the treatment process, they should continue the course.

Another unique aspect of our case is that our patient had a known history of soft drusens. Several clinical trials of FGFR inhibitors specifically excluded patients with known retinal pathology including soft drusens. It is not known whether or not the FIGHT-202 trial for pemigatinib included patients with drusen in their study, as their exclusion criteria included patients with ‘clinically significant retinal disorders.’ The importance of preexisting RPE alterations is likely highlighted in the proposed mechanism of SRF development in FGFR- and MEK-induced retinopathy. As FGFR acts upstream of MEK kinase in the FGF–MAPK pathway, similarities have been reported in their presentations and development (8, 11). Tyagi et al. noted that MEK inhibitors causing thickening of the ellipsoid zone due to swelling of the photoreceptors, and granular deposits with increased autofluorescence is suggestive of abnormal lipofuscin clearance due to the RPE dysfunction (12). These changes persisted despite the discontinuation of chemotherapeutic treatment. The presence of hypofluorescent foci on FAF suggestive underlying RPE pathology has also been noticed in MEK retinopathy. Additionally, electrooculogram (EOG) abnormalities and anti-RPE antibodies have been reported in patients with serous SRF in MEK retinopathy, implicating RPE pump function failures as the likely etiology for the accumulation of SRF (13). It is unclear at present if the presence of drusen can have any impact on the RPE in a way that may predispose patients to developing FGFR-induced SRF. At present, no longitudinal criteria for monitoring FGFR-induced SRF has been established (14). FAF may still be appropriate to establish a baseline exudative presentation and to differentiate from other causes of central retinopathy.

Though there are similarities between FGFR inhibitors and MEK inhibitors, FGFR does have a larger reach of influence, as it can impact PI3K pathways and intracellular calcium signaling (11). Additionally, though many cases, as well as our own, have had resolution of SRF following discontinuation of the medication, other reports have noted retained damage to the RPE (12). The MEK inhibitor lacnotuzumab, which functions upstream of other MEK inhibitors, has been associated with a prolonged course with a lack of improvement following discontinuation (15). It may be beneficial to perform serial OCT studies and observe the RPE’s function and resolution in patients with suspected risk factors. Additionally, more cases need to be identified and investigated to establish a correlation between drusen or other retinal disorders as risk factors for developing FGFR inhibitor-induced SRF. Importantly, every case should be individualized with the collaboration of the patient’s oncologist with the aim to improving the patient’s overall and ocular health.

## Data availability statement

The original contributions presented in the study are included in the article/supplementary material. Further inquiries can be directed to the corresponding author.

## Ethics statement

Written informed consent was obtained from the individual(s) for the publication of any potentially identifiable images or data included in this article.

## Author contributions

DB-A contributed collection of OCT imaging, multiple patient examinations, writing of document and editing. GJ contributed collection of OCT imaging, multiple patient examinations, writing of document and editing. RL contributed to editing of the paper. All authors contributed to the article and approved the submitted version.
